# Methane emission and sulfide levels increase in tropical seagrass sediments during temperature stress: A mesocosm experiment

**DOI:** 10.1002/ece3.6009

**Published:** 2020-02-05

**Authors:** Rushingisha George, Martin Gullström, Matern S. P. Mtolera, Thomas J. Lyimo, Mats Björk

**Affiliations:** ^1^ Department of Ecology, Environment and Plant Sciences Seagrass Ecology and Physiology group Stockholm University Stockholm Sweden; ^2^ Tanzania Fisheries Research Institute (TAFIRI) Dar es Salaam Tanzania; ^3^ Department of Biological and Environmental Sciences University of Gothenburg Kristineberg, Fiskebäckskil Sweden; ^4^ Institute of Marine Sciences (IMS) University of Dar es Salaam Zanzibar Tanzania; ^5^ Department of Molecular Science and Biotechnology University of Dar es Salaam Dar es Salaam Tanzania

**Keywords:** biogeochemical processes, coastal sediment, methane, ocean warming, photosynthetic performance, sulfide, tropical seagrass, Western Indian Ocean

## Abstract

Climate change‐induced ocean warming is expected to greatly affect carbon dynamics and sequestration in vegetated shallow waters, especially in the upper subtidal where water temperatures may fluctuate considerably and can reach high levels at low tides. This might alter the greenhouse gas balance and significantly reduce the carbon sink potential of tropical seagrass meadows. In order to assess such consequences, we simulated temperature stress during low tide exposures by subjecting seagrass plants (*Thalassia hemprichii*) and associated sediments to elevated midday temperature spikes (31, 35, 37, 40, and 45°C) for seven consecutive days in an outdoor mesocosm setup. During the experiment, methane release from the sediment surface was estimated using gas chromatography. Sulfide concentration in the sediment pore water was determined spectrophotometrically, and the plant's photosynthetic capacity as electron transport rate (ETR), and maximum quantum yield (Fv/Fm) was assessed using pulse amplitude modulated (PAM) fluorometry. The highest temperature treatments (40 and 45°C) had a clear positive effect on methane emission and the level of sulfide in the sediment and, at the same time, clear negative effects on the photosynthetic performance of seagrass plants. The effects observed by temperature stress were immediate (within hours) and seen in all response variables, including ETR, Fv/Fm, methane emission, and sulfide levels. In addition, both the methane emission and the size of the sulfide pool were already negatively correlated with changes in the photosynthetic rate (ETR) during the first day, and with time, the correlations became stronger. These findings show that increased temperature will reduce primary productivity and increase methane and sulfide levels. Future increases in the frequency and severity of extreme temperature events could hence reduce the climate mitigation capacity of tropical seagrass meadows by reducing CO_2_ sequestration, increase damage from sulfide toxicity, and induce the release of larger amounts of methane.

## Graphical abstract

In order to estimate responses to warming in seagrass sediments, a mesocosm experiment with tropical seagrasses was performed. We measured methane release, sulfide concentration in the sediment pore water, and the plant's photosynthetic capacity. The higher temperatures had clear negative effects on the photosynthetic performance of seagrass plants and, at the same time, a clear positive effect on methane emission and the level of sulfide in the sediment. Future increases in the frequency and severity of extreme temperature events could hence cause tropical seagrass meadows to suffer increased damage from sulfide toxicity and induce the release of larger amounts of methane.

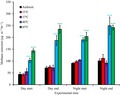

## 1 INTRODUCTION

Methane (CH_4_), after CO_2_ the most important of greenhouse gases, is to a large degree emitted from wetlands, which can contribute as much as 30%–50% of the global emissions (Bridgham, Cadillo‐Quiroz, Keller, & Zhuang, 2013; Laanbroek, 2009; Stocker et al., 2013; Whiting & Chanton, 1993). How seagrass systems might contribute to these emissions has received comparably little attention, although valuable studies have been published (Bahlmann et al., 2015; Barber & Carlson, 1993; Deborde et al., 2010; Garcias‐Bonet & Duarte, 2017; Oremland, 1975). Temperature increases have been shown to enhance methane emissions from freshwater systems (Yvon‐Durocher, Hulatt, Woodward, & Trimmer, 2017; Yvon‐Durocher, Montoya, Woodward, Jones, & Trimmer, 2011), and recently, it has been shown that methane emission from seagrass meadows rises substantially when seagrasses are disturbed (Burkholz, Garcias‐Bonet, & Duarte, 2019; Lyimo et al., 2017), and based on calculations of methane emission in seagrass sediments from the Red Sea, it has been suggested that the present estimations of methane emissions from natural systems might have to be increased by about 30% to account for hitherto unrecognized contributions from seagrass systems (Garcias‐Bonet & Duarte, 2017). In general, the methane production of biological systems is closely correlated with the productivity of the plants within the system (Borges, Speeckaert, Champenois, Scranton, & Gypens, 2018; Bridgham et al., 2013), and for wetlands in particular, there is a clear positive correlation between emission of methane and net ecosystem production (Whiting & Chanton, 1993). This has been explained by the results from C14‐labeling studies showing that methane production in wetlands is partly driven by recent plant photosynthates such as root exudates (Dorodnikov, Knorr, Kuzyakov, & Wilmking, 2011; King & Reeburgh, 2002; Megonigal et al., 1999). In a study focusing on tundra, it was shown that approximately 2%–3% of the carbon fixed by photosynthesis at the peak of the growing season was subsequently emitted as methane, which means that nearly 75% of the methane emissions from the tundra ecosystem may originate from carbon recently fixed in photosynthesis (King & Reeburgh, 2002).

The methanogenic microbial community of sediments and soils appears to be sometimes coexisting with the sulfate‐reducing community (Oremland & Taylor, 1978; Pender et al., 2004; Sanz‐Lázaro, Valdemarsen, Marín, & Holmer, 2011; Van Bodegom & Stams, 1999), where methane production and sulfate reduction can take place simultaneously in anoxic sediments (Oremland, Marsh, & Polcin, 1982; Oremland & Taylor, 1978; Van Bodegom & Stams, 1999). In addition, the sulfide production in the seagrass sediment is linked to plant productivity (Barber & Carlson, 1993), as oxygen input (via the lacunae) into the sediment of the rhizosphere as radial oxygen loss (ROL) can oxidize the sediment and result in the suppression of both sulfide and methane production (Borum, Sand‐Jensen, Binzer, Pedersen, & Greve, 2007; Devereux et al., 2011; Laanbroek, 2009; Marbà et al., 2010). In temperate seagrasses, a significant release of oxygen from the roots to the rhizosphere occurs (Frederiksen & Glud, 2006; Jensen, Kühl, Glud, Jørgensen, & Priemé, 2005), protecting the plants against sulfides and other toxins (Brodersen, Nielsen, Ralph, & Kühl, 2015; Pedersen, Borum, Duarte, & Fortes, 1998) by mediating the conversion of these toxic compounds to less toxic forms through the activity of aerobic micro‐organisms or by chemical oxidations in the oxidized rhizosphere (Brodersen et al., 2018; Laanbroek, 2009; Sanz‐Lázaro et al., 2011). Root‐derived dissolved organic carbon (DOC) has been shown to be a major sink of photosynthesized carbon in seagrasses (*Thalassia hemprichii* and *Enhalus acoroides*), and when it is excreted from the root system to the sediment, it stimulates the activity of micro‐organisms around the seagrass roots (Jiang et al., 2018). In addition, it has been shown in terrestrial systems that such photosynthates from plants may serve as a substrate for methanogenic microbes and end up in the methane emitted from the system (Dorodnikov et al., 2011; King & Reeburgh, 2002).

Water temperature is one of the most important factors affecting productivity of seagrass plants, where high temperatures have been shown to negatively affect photosynthetic performance of several tropical seagrasses (Bulthuis, 1987; Campbell, McKenzie, & Kerville, 2006; Collier & Waycott, 2014; Hurd, Harrison, Bischof, & Lobban, 2014; Lee, Park, & Kim, 2007). In tropical intertidal seagrass habitats, the water temperature is highly influenced by contemporary tidal regimes, where high diurnal temperature changes are common (Bridges & McMillan, 1986; Burdick, Dionne, Boumans, & Short, 1996; Koch & Erskine, 2001; Pedersen, Colmer, Borum, Zavala‐Perez, & Kendrick, 2016). During daytime, and especially in spring low tide, seagrasses within the intertidal areas of Zanzibar, Tanzania, frequently experience elevated water temperatures of 40–44°C for periods of 3–4 hr, which have clear and immediate negative effects on the photosynthetic performance of the seagrass plants (George, Gullström, Mangora, Mtolera, & Björk, 2018). The frequency and intensity of temperature stress events are anticipated to increase in the future under human‐driven climate change (Bernstein et al., 2008; Pachauri et al., 2014) and are thus likely to exacerbate the negative impacts of warming on tropical seagrass meadows. Therefore, an improved understanding of the response of temperature stress on biogeochemical processes in sediment and its linkage to plant productivity could result in better predictions of ocean warming impacts on carbon sequestration and storage in tropical seagrass meadows.

In the present study, we carried out a mesocosm experiment to determine the effects of temperature stress on methane emission and sulfide levels in tropical seagrass sediment, and whether these processes relate to the photosynthetic performance of seagrass plants. We hypothesized that (a) reduction in photosynthetic performance by higher water temperature will increase methane emission and the sulfide pool of the sediment, (b) since effects by temperature on the photosynthetic performance of seagrass are shown to be immediate, the effects of increased temperature on methane emission and the sulfide pool in the sediment will react directly and increase with days of repeated stress, and (c) the changes in sediment processes will not be large enough to reduce the sedimentary organic carbon content.

## 2 MATERIALS AND METHODS

### 2.1 Plant material and associated sediment

Square sods (0.4 × 0.4 m) of *Thalassia hemprichii* (Ehrenberg) Ascherson, a commonly distributed seagrass species in the WIO region (Green & Short, 2003; Gullström et al., 2002; Short, Carruthers, Dennison, & Waycott, 2007), were carefully (without disturbing their structure) collected at three separate times (three days before the start of each experiment) between February and March 2015 from the Mbweni area, Unguja Island (Zanzibar), Tanzania (6°21′S, 39°20′E). In the collection site, many seagrass species grow in the intertidal area, where the water temperature is occasionally heated up to 40°C and above in short pulses (George et al., 2018). Prior to the experiment, we estimated seagrass shoot density at the collection site, which was 1,032 ± 47 shoots/m^2^ (mean ± *SE*). Seagrasses were collected using a 25 cm^2^ and 30 cm deep stainless steel corer, which was gently pressed into the sediment, permitting a large sod of seagrass to be cautiously lifted out, representing the shoot density of the collection area. The sods were carefully deployed into five separate 100 L plastic containers (referred to as small containers), and seawater was gradually added up to the 80 L level. The containers were immediately transported to the experimental site (Buyu, the experimental facility of the Institute of Marine Sciences, University of Dar es Salaam; 6°26′S, 39°23′E) located 7 km south of the collection site. At the experimental site, the small containers (with seagrass sods) were deployed in five separate 400 L white plastic tanks (referred to as large containers) with seawater (filled below the rim of the small containers) to offset against undesirable temperature fluctuations. The 100 L containers (with seagrass sods) were filled with seawater up to 80 L and bubbled by electrical air pumps to facilitate water mixing. The containers were exposed to different temperature treatments following the description below (see Section 2.2). Prior to the start of an experimental run, the plants were allowed to acclimatize for three days.

### 2.2 Experimental setup

The experiment was conducted outdoors under ambient light and photoperiod conditions, from 28 January to 20 March 2015, in the northeast monsoon, when seagrasses in the region commonly experience steady conditions with high average water temperatures (George et al., 2018). Due to equipment limitations and logistical constraints (i.e., the time taken to perform measurements with available equipment), the experiment could not be replicated simultaneously; instead, the full setup was repeated three times (approximately every second week) with new plant material and water. The weather conditions were similar throughout the three experimental runs (with no extreme weather events), thus making the three experimental runs comparable while still sustaining natural variability in, for example, light and temperature. In each experimental run, different sods of seagrasses were exposed to either control (ranging from 29 to 33°C, average 31°C), 35, 37, 40, or 45°C. The heat stress was applied daily for 3 hr, from 10:00 to 13:00, to simulate temperature stress during daytime low tidal exposure (cf. Figure 2) for seven consecutive days, by heating the water in the small containers with submersible thermostatic heaters until the desired temperatures (i.e., 35, 37, 40, and 45°C) were reached (after up to 2 hr). After the heat stress, the small containers were gradually drained until reaching 75% of the original water level, and subsequently refilled with new seawater of ambient temperature in order to lower the experimental temperatures to ambient levels (to simulate a returning high tide). The temperature treatment levels were chosen based on available field data recorded during the northeast monsoon (George et al., 2018), previous experimental works on tropical seagrasses (Campbell et al., 2006; Collier & Waycott, 2014) and the expected increase in sea surface temperature under a projected global warming scenario by the year 2100 (Bernstein et al., 2008; Pachauri et al., 2014). The ambient containers were also partially drained, with 75% of the water being removed and exchanged with new seawater once per experimental run (on the third day) to avoid effects of evaporation and nutrient limitation. This setup allowed us to compare responses between the ambient water temperature and the temperature elevation runs simultaneously.

### 2.3 Measurements of water temperature and light

To assess the natural variability in water temperature and light within the seagrass canopy, combined temperature and light loggers (HOBO Pendant Temp/Light Logger 8K; Onset) were attached among seagrass shoots (about 10 cm above the sediment), during February and March, in the location where the seagrasses were collected. The loggers recorded water temperature (°C) and light (lux) every 30 min. Loggers were also installed in a similar way in each treatment of the experimental setup. Data from field loggers were retrieved after 31 days, while in the experimental setup, logged data were retrieved after seven days of each experimental run. The light measurements recorded by the loggers were converted into µmol photons m^−2^ s^–1^ by calibrating the light logger against a PAR sensor (Model IL 1400A photometer; International Light Technologies).

### 2.4 Assessment of photosynthetic performance

The effects of elevated temperatures on seagrass photosynthetic performance were assessed from chlorophyll fluorescence measurements of electron transport rate (ETR) and maximum quantum yield (Fv/Fm) of photosystem (PS) II using a pulse amplitude modulated (PAM) fluorometer (Diving PAM Walz). The basic parameters of chlorophyll fluorescence (Fo, F, Fm, Fm′) were measured at midday hours (from 12:00 to 13:00) on the middle part (5 cm from the leaf base) of the third youngest leaf, on three replicate leaves (during the first, fourth, and seventh days of the different experimental runs). Similarly, measurements of Fv/Fm were performed at midnight hours (from 24:00 to 1:00) in all experimental treatments. The ETR was estimated as described by Beer, Björk, Gademann, and Ralph (2001) by multiplying the effective quantum yield (ΔF/Fm′) by the photosynthetic photon flux density (PPFD) absorbed by the leaf (the absorption factor, AF) and by 0.5 (assuming equal distribution of photons absorbed by PSI and PSII). The absorptance factor (AF) was determined by the method described by Beer and Björk (2000) using eight replicate leaves (using the middle of the third youngest leaf). The light sensor was fixed on a solid surface pointing upwards; the middle part of the third youngest leaf was then placed over the light sensor of the diving PAM, and incident PPFD at saturation was recorded with and without leaf. Thus, an average absorption factor for the leaves of *Thalassia hemprichii* of 0.722 ± 0.001 was obtained.

### 2.5 Estimation of pore‐water sulfide concentration

Pore‐water sulfide samples were collected at three different sediment depths (5, 10, and 15 cm) from all experimental treatments in daytime (from 12:00 to 13:00) and at night (from 00:00 to 1:00) during the first and last (day 7) days of the experiment. A particularly adapted needle (20 cm in length and with a diameter of 0.2 cm), connected to a 60 ml syringe (10 cm long hand‐held) by a rubber tube (15 cm long and 0.2 cm in diameter), was used to draw pore water from separate sediment depths. Approximately 10 ml of pore water was drawn from the sediment, of which 5 ml was filtered using syringe microfilters (GF/F 0.2 µm; Sigma‐Aldrich) and injected into a tightly closed 100 ml conical flask filled with 10 ml of 2% zinc acetate (w/v) to prevent sulfide oxidation. In the laboratory, each sample was added up with 40 ml of distilled water, followed by 5 ml of dimethyl‐paraphenylene diaminesulfate (DPDS), and immediately thereafter, 0.25 ml of the ferric ammonium sulfate (10%) solution was added and mixed vigorously. The reaction of this mix leads to blue coloration, which indicates presence of sulfide. The dissolved sulfide concentration in the mixture was determined spectrophotometrically at 663 nm and calculated by comparing to a standard curve of known sulfide concentration, as described in Lawrence, Davis, and Compton (2000).

### 2.6 Estimation of methane emission

Gas samples for emission estimations were collected using Perspex chambers (30 cm long × 4 cm in inner diameter) at the start and end of the experiment, and during both day (10:00 to 13:00) and night (22:00 to 1:00). One chamber was gently pushed into the sediment to approximately 15 cm depth in each experimental treatment. The chamber covered an area of 0.0013 m^2^ and had an internal volume of 0.38 L. Chambers were then tightly closed with rubber stoppers (to 2 cm inside the chamber from the top) to contain a 7 cm air phase above the 6 cm water phase inside the chambers, and subsequently incubated for 3 hr. Six 1 ml gas samples were collected from the air phase from each chamber, using 1 ml airtight syringes, at both the beginning and end of the incubation. The syringes with gas samples were inserted into a rubber stopper and kept at room temperature (i.e., 25°C) for 3–4 days before analysis (Lyimo et al., 2017; Lyimo, Pol, & Op den Camp, 2002). The gas samples were then analyzed using a gas chromatograph (HP‐5890 series II, Hewlett Packard) equipped with a flame ionization detector (GC‐FID) and a HayeSep Q (60–80 mesh) column. The carrier gas was nitrogen at a flow rate of 40 ml/min; oven‐, detection‐, and injection‐port temperatures were 100, 175, and 175°C, respectively. The samples were analyzed at the Department of Molecular Biology and Biotechnology, University of Dar es Salaam.

The emission of methane from the water was then calculated as in Lyimo et al. (2002). In summary, we took the amount of methane after 3 hr (minus the ambient atmospheric amount of methane) minus the methane amount at time 0 (minus the ambient atmospheric amount of methane) to obtain the change over time. The concentration in µmoles of the samples was obtained from a linear equation generated from standard gas samples and recalculated as units over time and area, to get µmol m^−2^ hr^−1^. By multiplying with the molar weight of methane (16 g/mol), we obtained the methane emission rates in µg m^−2^ hr^−1^.

### 2.7 Estimation of organic matter (OM) content

Sediment samples for estimation of OM content were collected from all experimental treatments using a 10‐cm‐long hand‐held syringe corer (open in one end) that extracted a volume of 60 ml at the start and end of the experiment. The samples were packed in separate plastic bags, kept in cool boxes, and transported to the laboratory. In the laboratory, sediment samples were transferred to separate empty porcelain dishes with known weight (MP) and dried in an oven at 60°C for 48 hr for determination of dry weight (MPDS). Then, the oven temperature was increased to 500°C for 48 hr to burn the soil into ash. After cooling, the ash samples and porcelain were weighed (MPA). The percentage OM content was calculated according to the formula below:

Determination of the mass of the dry soil:

(1) MD=MPDS-MP

Determination of the mass of the ashed soil:

(2) MA=MPA-MP

Determination of the mass of OM:

(3) MO=MD-MA

Determination of the percentage OM content:

(4) OM=MO/MD×100

### 2.8 Data analysis

Effects of elevated midday temperature stress on plant photosynthetic performance (i.e., ETR and Fv/Fm) and methane emission among experimental treatments were analyzed by one‐way analysis of variance (ANOVA). T tests were performed to compare elevated temperature treatments with the ambient temperature conditions. Two‐way ANOVA was used to test the effects of time and sediment depth (as fixed factors) on sulfide. Prior to the analyses, homogeneity of variance was checked using Levine's test, and as there was no heterogeneity found, all analyses were performed on raw data. Multiple linear regression analysis was used to explore the relative importance of temperature and ETR on sulfide levels and methane emission. All data analyses were carried out using Statistica v. 13.

## 3 RESULTS

### 3.1 In situ daily maximum water temperature and underwater irradiance

**Figure 1:**
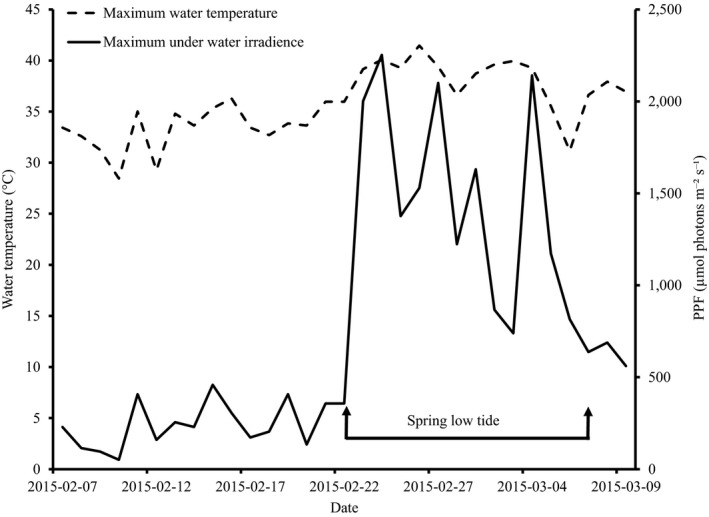
In situ daily maximum water temperatures and underwater irradiance recorded on top of the seagrass canopy from February to March 2015, at the seagrass collection site in Mbweni, Zanzibar. Data are from in situ loggers

**Figure 2:**
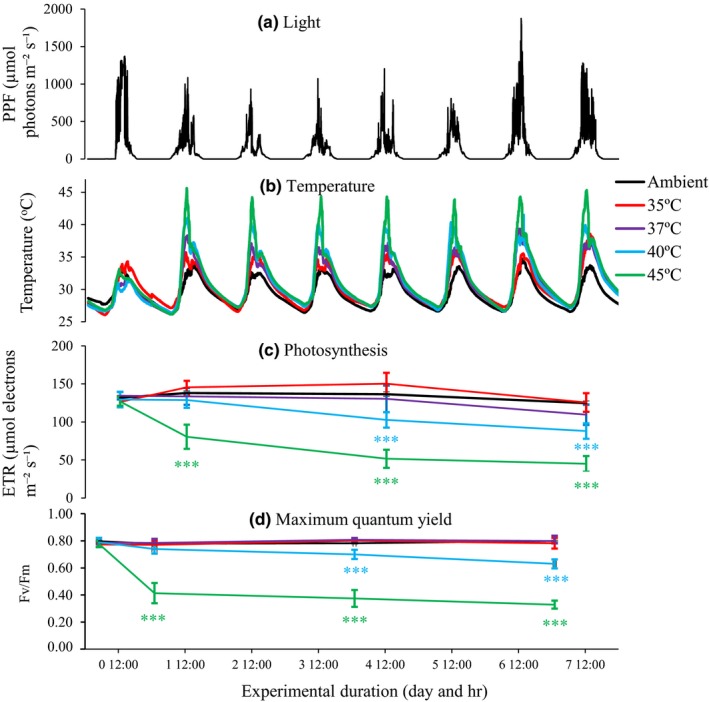
Electron transport rate (ETR) (c) and maximum quantum yield (Fv/Fm) (d) measured in shoots of the tropical seagrass *Thalassia hemprichii* when exposed under different temperature levels (b) and ambient light (a). Error bars show means ± *SE* (*n* = 3). Asterisks (***) indicate significant differences (based on *t* tests) in electron transport rate (c) and Fv/Fm (d) between elevated temperature treatments for each experimental time and the ambient conditions at *p* < .001. The start levels of Fv/Fm were 0.797 ± 0.02 at ambient conditions, 0.773 ± 0.02 at 35°C, 0.787 ± 0.01 at 37°C, 0.789 ± 0.03 at 40°C, and 0.777 ± 0.02 at 45°C

The maximum water temperatures within the seagrass canopy occurred during low tides and ranged from 28.5 to 41.5°C. During the majority of days (61%), the maximum water temperature exceeded 35°C (Figure 1). The maximum light levels were mostly between 100 and 1,000 µmol photons m^−2^ s^−1^, with 26% of the days reaching above 1,500 µmol photons m^−2^ s^−1^ and coinciding with spring low tides (Figure 1). General light levels in the experiment were within the range of in situ values (Figure 2a). The selected temperature levels were also within the range of natural values, but with the highest temperature treatment (45°C) being slightly higher than the maximum temperature recorded in situ (Figure 2b).

### 3.2 Photosynthetic performance

The photosynthetic rate, ETR, already became clearly reduced in the 45°C treatment during the first day of the experiment (from day 0 to day 1) (Figure 2c; *t* test, *p* < .05). The ETR then remained lower in both the 40 and 45°C treatments throughout the experiment (Figure 2c; *t* test, *p* < .05). There were no significant changes in the other temperature treatments during the experiment. The maximum quantum yield, Fv/Fm, was significantly affected in the 45°C treatment during day 1, day 4, and day 7 as well as in the 40°C treatment during day 4 and day 7 (Figure 2d; *t* test, *p* < .05), with a reduction in Fv/Fm from 0.8 to 0.3–0.6 in the high temperature (i.e., 40 and 45°C) treatments over the duration of the experiment, as compared with no change in the ambient, 35 and 37°C temperature treatments (Figure 2d).

### 3.3 Sediment sulfide concentration

**Figure 3:**
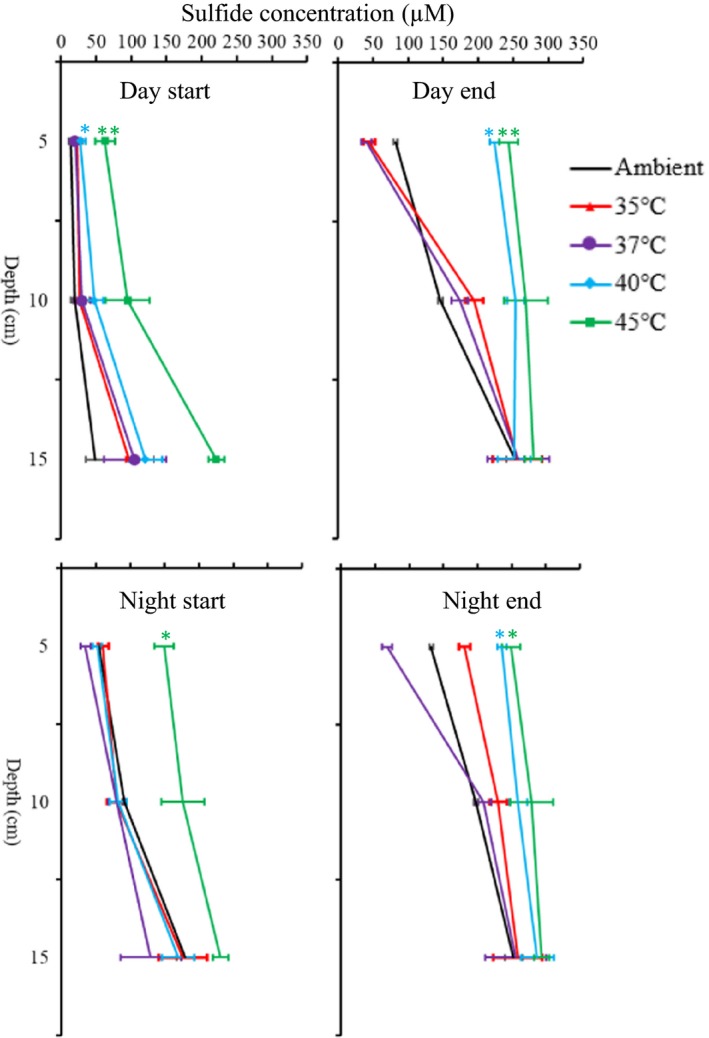
Profiles of pore‐water sulfide concentration in sediment with respect to elevated midday temperature stress collected in daytime and at night at the start and end of the experiment. Error bars show means ± *SE* (*n* = 3). Asterisks indicate significant differences (based on *t* tests) in sulfide concentration in the surface sediment layer (5 cm depth) between elevated temperature treatments for each experimental time and the ambient conditions at *p* < .05 (*), *p* < .001 (**) or *p* < .001 (***)

Compared with the ambient temperature conditions in the upper surface layers (5 cm), sulfide levels differed significantly in the 40°C (Figure 3, day start, *t* test, *p* < .05; day end, *t* test, *p* < .05; night end, *p* < .05) and 45°C (Figure 3, day start, *t* test, *p* < .01; day end, *t* test, *p* < .01; night start *p* < .05; night end, *p* < .05) treatments within each experimental time. At the end of the experiment, we observed an increase from around 100 μM sulfide to above 200 μM sulfide in the surface sediment layers (0–5 cm) when comparing ambient and high temperature (40 and 45°C) treatments (for both day and night measurements). In all experimental conditions, the levels of sulfide were significantly higher during night than during daytime for both the start and end of the experiment (two‐way ANOVA, *p* < .001 for both start and end; Figure 3). Generally, the sulfide concentration increased with depth in all experimental conditions (two‐way ANOVA, *p* < .001, Figure 3).

### 3.4 Methane emission

**Figure 4:**
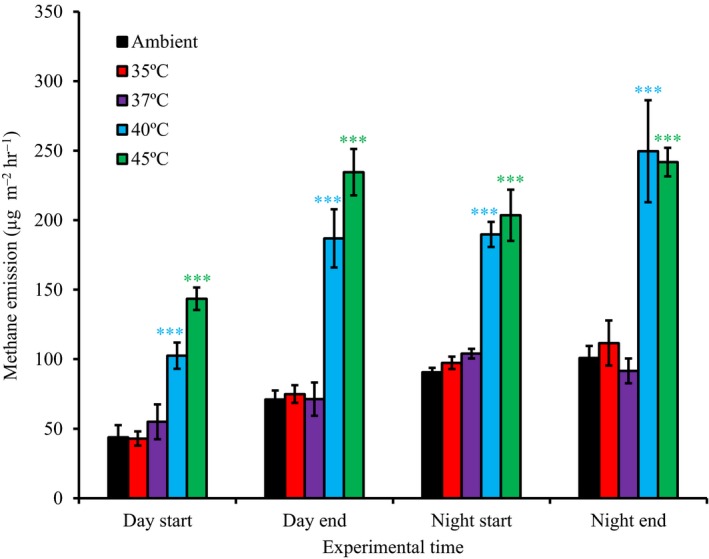
Methane emission from sediment taken from meadows dominated by the tropical seagrass *Thalassia hemprichii* measured during day and night at both the start and end of the experiment. Error bars are means ± *SE* (*n* = 3). Asterisks (***) above bars indicate significant differences (based on *t* tests) in methane emission between elevated temperature treatments for each experimental time and the ambient conditions at *p* < .001

Compared with the ambient methane emission levels, only the 40°C (Figure 4; day start, *t* test, *p* < .001; day end, *t* test, *p* < .001; night start, *t* test, *p* < .001; night end, *t* test, *p* < .001) and 45°C (Figure 4; day start, *t* test, *p* < .001; day end, *t* test, *p* < .001; night start, *t* test, *p* < .001; night end, *t* test, *p* < .001) treatments differed significantly during the different experimental times, but no other treatments did. Generally, methane emissions more than doubled, from 40–100 µg m^−2^ hr^−1^ to 100–250 µg m^−2^ hr^−1^ when comparing ambient and high temperature (40 and 45°C) treatments. In all experimental conditions (except for the 45°C treatment at the end of the experiment), methane emission was significantly higher at night than during the daytime for both the start and end of the experiment (*t* test, *p* < .01; Figure 4).

### 3.5 Organic matter content of the sediment

**Figure 5:**
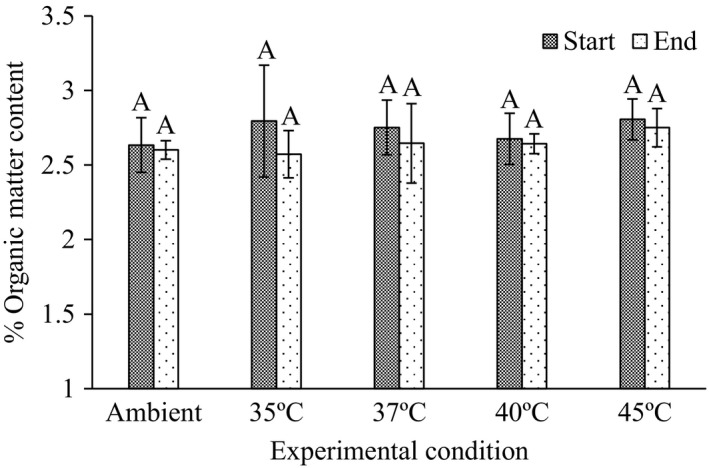
Percentage (%) organic matter (OM) content in sediment taken from meadows dominated by the tropical seagrass *Thalassia hemprichii* at the start and end of the experiment. Letters (A) denote no significant differences between means of OM (based on *t* tests) at *p* < .05

The average percentage OM content in the different experimental treatments ranged from 2.50% to 2.86% and did not vary significantly during the experimental period (Figure 5).

### 3.6 Relative importance of temperature and ETR on methane emission and the sulfide pool

**Figure 6:**
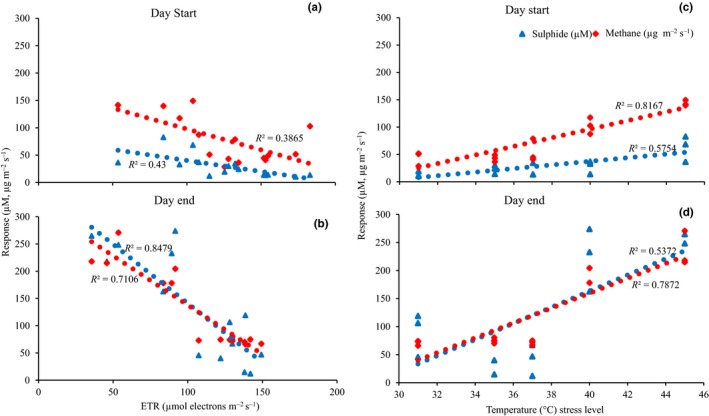
Relationships between electron transport rate (ETR) and temperature as predictor variables and methane (CH_4_) and sulfide (H_2_S) as response variables in daytime at the start (a, c) and end of the experiment (b, d). Dotted lines show significant correlations at *p* < .05

Both the sulfide concentration and methane emission were negatively correlated with ETR (Figure 6a,b) and Fv/Fm (not shown), whereas they were positively correlated with temperature (Figure 6c,d). In specific, the sulfide concentration and methane emission were strongly related to temperature in the beginning of the experiment (Table 1). In contrast, ETR showed a stronger relationship with both response variables in the end of the experiment (Table 1), while temperature (although weaker) was also related to methane emission in the end of the experiment (Table 1).

**Table 1:** Results from multiple linear regression analyses of temperature (temp) and ETR as predictor variables on sulfide concentration and methane at the start and end of the experiment. Significant values are shown in bold and probability (*p*) levels by stars (*p < .05, **p < .01, ***p < .001). β = standardized beta (regression coefficient), Adj *R* ^2^ = adjusted *R* ^2^

Dependent variable	Predictor variables				Overall model performance		
	β temp	*p* temp	β ETR	*p* ETR	Adj *R* ^2^	*F*	*p*
Sulfide concentration at the start	**0.57**	*	−0.33		.59	10.96	**
Sulfide concentration at the end	0.12		**−0.75**	*	.67	15.06	***
Methane emission at the start	**0.82**	***	−0.15		.80	29.60	***
Methane emission at the end	**0.40**	*	**−0.59**	**	.88	53.36	***

## 4 DISCUSSION

This study clearly shows that an increase in water temperature over a certain threshold resulted in positive effects on methane emission and the level of sulfide in the seagrass sediment and negative effects on the photosynthetic performance of seagrass plants. The effect observed by temperature stress was immediate (occurring during the first hours) and seen in all response variables—including methane emission, sulfide levels, ETR, and Fv/Fm. As indicated by the multiple regression analyses, the increases in methane and sulfide were at the start of the experiment foremost related to temperature, but at the end of the experiment (i.e., after seven days), the factor that could explain most of the increase in methane and sulfide was the photosynthetic performance (in terms of ETR) of the seagrass plants. Both the methane emission and the size of the sulfide pool were correlated with changes in ETR and temperature already during the first day, and with time, the correlations with ETR became stronger. It thus appears that there is some kind of connection between the decrease in photosynthetic oxygen production and the increases in methane and sulfide levels. However, this would probably be paralleled with direct effects of temperature on the methanogens within the sediment (as sediment‐associated methanogens may strongly affect temperature; Yvon‐Durocher et al., 2014).

The stress responses recorded upon the plants were similar in magnitude to a previous study on the same species (George et al., 2018), but with a slightly stronger influence at 40°C, as indicated in the ETR and Fv/Fm levels. The negative effects of high midday temperature stress on the photosynthetic performance were intensified by the number of repeated days of exposure, indicating chronic damage in the photosynthetic machinery as the maximum quantum yield (Fv/Fm) could not recover even after several hours of darkness and ambient temperatures (Beer, Björk, & Beardall, 2014; Hanelt, 1992; Maxwell & Johnson, 2000). The rise in methane emission concomitant with the decrease in photosynthetic capacity could be explained by a combination of effects on the microbial community in the sediment. The level of methane emission from a system is not only governed by the production (methanogenesis), it is equally important how much of the methane is oxidized (methanotrophy) (Bridgham et al., 2013; Dunfield, Knowles, Dumont, & Moore, 1993). Thus, the emission rates reported in this study are net emissions, which represent a balance between production and consumption of the whole system. In wetlands, a clear positive correlation between emission of methane and net ecosystem production has been documented (Borges et al., 2018; Bridgham et al., 2013; Whiting & Chanton, 1993; Yvon‐Durocher et al. 2011). It has also been shown that the methane production in the rhizosphere of wetland and tundra plants is to a large extent driven by recent plant photosynthates in the form of root exudates (Dorodnikov et al., 2011; King & Reeburgh, 2002; Megonigal et al., 1999). On the other hand, the photosynthetically derived oxygen that is transported to the rhizosphere by the roots of wetland plants (i.e., radial oxygen loss) can also suppress methane production (Laanbroek, 2009), and in temperate seagrasses, such a release of oxygen to the rhizosphere has been shown to also protect against sulfides and other toxins (Brodersen et al., 2015, 2018; Pedersen et al., 1998). Methanogenesis is also a temperature‐dependent process (Dunfield et al., 1993; Sanz‐Lázaro et al., 2011; Van Bodegom & Stams, 1999; Westermann, Ahring, & Mah, 1989; Zeikus & Winfrey, 1976), and rapid changes in temperature can result in simultaneous changes in methane production (Borges et al., 2018; Chin, Lukow, & Conrad, 1999; Høj, Olsen, & Torsvik, 2008; Segers, 1998; Van Bodegom & Stams, 1999). This is partly due to a direct effect on the process, where the methane production can have a Q_10_ (i.e., a relative increase in activity after an increase in temperature of 10°C) of 1.3–28 (Dunfield et al., 1993; Segers, 1998; Van Hulzen, Segers, Bodegom, & Leffelaar, 1999) and partly because of a temperature‐driven shift in the composition and activity of the microbial community (Conrad, Klose, & Noll, 2009; Høj et al., 2008; Pender et al., 2004).

A direct temperature effect on the production process could explain the immediate effect on methane production observed in the present study at the first temperature pulse, an effect similar to what has been observed in rice paddy soils (Van Bodegom & Stams, 1999); however, it would not be the only explanation to the response since the elevated methane emission was persistent, and even higher, at night, that is, after that the temperature had gone down to ambient levels. Furthermore, such a direct temperature response would be expected to follow the temperature increase more or less linearly (Nedwell & Watson, 1995; Van Hulzen et al., 1999; Yvon‐Durocher et al., 2014), which was not the case in our study. The results from our multiple regression analysis suggest a combination of direct temperature effects and secondary effects through the inhibition of photosynthetic performance, with the indirect temperature effects being stronger in the end of the experiment. Reasonably, effects on sulfide and methane through reduced photosynthetic rates would take longer to appear than direct temperature effects on methanogenesis. The seagrass root system and its effect on sediment oxygenation has shown to have clear effects on the composition of the surrounding microbial community (Jensen, Kühl, & Priemé, 2007), and it might thus be that the elevated temperature in our study both had a direct effect on the methanogens in the sediments and also altered the balance between the production and breakdown of methane by changing the oxygenation of the sediment, possibly leading to a shift in the composition of the sediment microbiome. The clear threshold of methane emission between 37 and 40°C corresponded with a distinct increase of sulfide levels above 37°C in the upper part of the sediment at the end of the experiment. The photosynthetic rate was the largest explanatory factor for the increases in both methane and sulfide at the end of the experiment, and as there were also clear negative correlations between ETR and both methane and sulfide, it seems like the raised levels of methane and sulfide in the end of the experiment were more closely related to the loss of photosynthetic capacity of the plants (causing changes in oxygen transport patterns and possibly buildup of dead organic matter) than a direct effect of temperature on the sediment processes.

The negative effects of temperature on photosynthetic rates increased with time, with the initial effects slightly stronger in the end of the experiment compared with the beginning, which was in line with the increase in both sulfide levels and methane emissions; in fact, the correlations of both sulfide levels and methane emission with the ETR became much stronger in the end of the experiment. The increases in sulfide levels were also markedly higher in the upper layers of the sediment (at 5 cm depth), indicating that the deeper anoxic zone extended upwards during the course of the study. The loss of photosynthetic capacity, and a subsequent decrease in the internal O_2_ transport to below‐ground tissues, has been shown to cause anoxia in seagrass sediments (Greve, Borum, & Pedersen, 2003; Nagel, 2007), and a decrease in photosynthetic capacity (by shading) of tropical seagrasses has been suggested to have a negative effect on the part of the microbial community of seagrass sediment using photosynthetically produced exudates from the seagrass (Barber & Carlson, 1993; Schrameyer et al., 2018). Such effects might have shifted the microbial activity to cause an additional increase in sulfate reduction and methanogenesis, causing the increase in sulfide levels and methane emissions observed in this study, possibly also strengthened by the decay of below‐ground tissues, which has been observed under events of elevated midday temperature stress (George et al., 2018) and suggested to be a source of the organic matter supporting sediment CH_4_ production in seagrass meadows (Barber & Carlson, 1993). As expected, any changes in the microbial activity and composition did, however, not result in any significant change in the organic matter content of the sediment.

Under future ocean warming conditions, increases in the frequency and severity of temperature spike events (at 40–45°C levels) are expected (Arias‐Ortiz et al., 2018; Pachauri et al., 2014). Under such a scenario, tropical seagrass meadows will suffer losses, and their productivity will be impaired. The results of the present study suggest that this will be followed by changes in greenhouse gas dynamics, and previous studies suggest that the effects can be scaled up. In a meta‐analysis of natural methane emissions from a wide range of ecosystems, it was found that seasonal variations in methane emissions in general exhibit a similar temperature dependence to the production of methane estimated from laboratory cultures of methanogens and anaerobic microbial communities (Yvon‐Durocher et al., 2014). The results from this study thus indicate that, in the future, tropical seagrass meadows could not only lose a substantial part of their carbon sequestration capacity but could also significantly increase their release of methane.

## CONFLICT OF INTEREST

The authors declare that the research was conducted in the absence of any commercial or financial relationships that could be construed as a potential conflict of interest.

## AUTHOR CONTRIBUTIONS

RG, MG, and MB contributed to experimental design. RG, MG, MSPM, TJL, and MB contributed to manuscript writing.

## Data Availability

Data used for analysis are presented in the paper and are available from the Dryad Digital Repository. ://doi.org/10.5061/dryad.xd2547dd2
